# Transactions of International Medical Congress of Philadelphia

**Published:** 1877-11

**Authors:** 


Transactions of the International Medical Congress of Philadelphia, 1876. Edited for the Congress. By John Ashurst, A.M., M.D. Philadelphia. 1877.

These transactions make a volume of over twelve hundred pages, four hundred of which are filled with eleven exceedingly interesting and useful addresses delivered before the Congress on medical subjects, and contributions from the various sections, numbering about sixty-nine interesting papers.

The fourth address was on surgery, and delivered by the late Paul F. Eve, whose death is noticed in this issue of the Journal. This was the last public act known of this remarkable man. His opening remark is so unique, and strikes us as so characteristic of this native Georgian, that we copy as follows:

"Gentlemen of the International Medical Congress:

"The language of the aborigines of America has ever been admired for its simplicity and significance. In the State of Mississippi, itself implying the father-of-waters, is a county called Itawambi, derived from the title given an Indian chief, upon whom all possible honors had been conferred, so that nothing more could be done for him. Standing here this day, the representative of the most demonstrative and efficient department of the healing art—the recipient, too, of many unmerited favors—fully conscious of the inability to do justice to the subject proposed, or satisfactorily improve the illustrious occasion—an event without precedent, and which no living man will ever see again—language fails to convey or words express what is now felt. With all the simplicity of the Indian, and the sincerity of the Christian, I would say to you, my professional brethren, who have done so much for me, *Itawambi*."

The Transactions, taken altogether, will be found unusually interesting and instructive.

